# Lady Cooks for Hospitals

**Published:** 1887-11-26

**Authors:** 


### The Editor's Letter-Box.

[Our Correspondents are reminded that prolixity is a great bar to publication, and that brevity of style and conciseness of statement greatly facilitate early publication.]

### LADY COOKS FOR HOSPITALS.

I think a "Resting Matron's" idea excellent; what an advantage a lady cook would be in a hospital! How valued she would be! She would at once raise the standard of honour in the kitchen, and would raise the whole tone. Would that a Florence Nightingale could see this wide field, and act as pioneer for the kitchen, as she did for the nursing part of the hospital. We all know how much of the comfort of a house radiates from the kitchen. If cook is "in a bad temper" the meal is spoiled, and the entire dinner a failure; the master of the house cross, and the mistress sad and troubled. Your correspondent "S." asks about lifting weights. No kitchen weight is as heavy as a helpless patient, no duty so repulsive as many of those in nursing. Any lady (in heart) might take the head of a kitchen, keep her maids in order as much as the lady nurse upstairs her probationers; might have her own private room, yes, and her piano too, and neither such heavy or trying work or the long hours of the lady nurse upstairs, and, to complete the picture, a much better salary. I would send my name to "S." as the first matron to hail the advent of a lady cook in the kitchen of the hospital to which I was matron, but, alas! I am now (John Brown as I read my letter to him)

A Married Matron.

